# Regulation of matrix metallo-proteinase expression by extracellular matrix components in cultured hepatic stellate cells

**DOI:** 10.1186/1476-5926-2-S1-S20

**Published:** 2004-01-14

**Authors:** Da-Ren Wang, Mitsuru Sato, Takeya Sato, Naosuke Kojima, Nobuyo Higashi, Haruki Senoo

**Affiliations:** 1 Department of Cell Biology and Histology, Akita University School of Medicine, Akita 010-8543, Japan

## Abstract

Hepatic stellate cells (HSC) changed their morphology and function including production of matrix metalloproteinases (MMPs) in response to extracellular matrix (ECM) component used as a substratum in culture. We examined in this study the regulatory role of ECM component on expression of MMPs and tissue inhibitor of metalloproteinase (TIMP) in rat HSCs cultured on polystyrene, type I collagen-coated surface, type I collagen gel, or Matrigel, respectively. When cultured on type I collagen gel, HSCs showed the asteroid cell shape and MMP-1 activity, as detected by in situ zymography. Expression of MMP-1 protein and mRNA were examined by using immunofluorescence staining and RT-PCR analysis in HSCs cultured on type I collagen gel. Active form of MMP-2 was detected by gelatin zymography in the conditioned medium of HSCs cultured on type I collagen gel, whereas it was not detected when HSCs were cultured on polystyrene, type I collagen-coated surface, or Matrigel. Increased MMP-2 mRNA was detected by RT-PCR in HSCs cultured on type I collagen gel. Increased MT1-MMP proteins were shown to localize on the cell membrane by using immunofluorescence staining in HSCs cultured on type I collagen gel. Elevated expression of membrane-type matrix metallproteinase-1 (MT1-MMP) mRNA and tissue inhibitor of metalloproteinase-2 (TIMP-2) mRNA was detected by RT-PCR in HSCs cultured on type I collagen-coated surface or type I collagen gel. These results indicate that expression of MMPs and TIMP-2 is regulated by ECM components in cultured HSCs, suggesting an important role of HSCs in the remodeling of liver tissue.

## Introduction

Extracellular matrix (ECM) plays important roles in maintaining tissue structure, development and proliferation [1]. The interaction of cells with ECM is essential for cell behavior. Remodeling of ECM by matrix metalloproteinases (MMPs), which can degrade most ECM components, plays a critical role in both physiological and pathological processes.

Hepatic stellate cells (HSCs) are located in the hepatic perisinusoidal spaces, and have multifunction [2,3]. Hepatic fibrosis, scar deposition in response to chronic injury, is similar in all forms of liver disease. Accumulation of fibrillar, or type I, collagen occurs in the subendothelial space between hepatocytes and endothelial cells, where it replaces a low-density basement membrane-like matrix containing type IV collagen. The accumulation of type I collagen has a direct effect on HSC activation through an unknown mechanism [4]. HSC activation is further accelerated by upregulation of MMP-2 activity, because MMP-2 degrades the normal subendothelial matrix, promoting its replacement by interstitial collagen [5]. The activity of MMP-2 is tightly regulated by specific inhibitors including tissue inhibitor of metalloproteinase-2 (TIMP-2) and by activators including membrane-type matrix metallproteinase-1 (MT1-MMP) [6]. Cultured HSCs have been found to change their morphology and function including the production of MMPs and ECM according to the ECM components used as a substratum. In this study we examined the regulatory role of ECM components used as substrata on expression of MMPs and TIMP-2 in cultured HSCs.

## Methods

HSCs were isolated from the rat liver, primary cultured, and then subcultured in Dulbecco's modified Eagle's medium containing 10% FBS using several substrata including polystyrene surface, type I collagen-coated surface, type I collagen gel, or Matrigel. HSCs cultured on polystyrene or on type I collagen gel were immunofluorescently stained for MMP-1 and MT1-MMP. Interstitial collagenase activity in HSC culture on type I collagen gel was detected by in situ zymography. Pro- and active-forms of MMP-2 in the conditioned medium were analyzed by gelatin zymography in HSCs cultured on several substrata. MT1-MMP proteins in cell lysates of HSCs cultured on several substrata were analyzed by Western blot analysis. Total RNA was isolated from each HSC culture for reverse transcription-polymerase chain reaction (RT-PCR) analysis to detect MMP-1, MMP-2, MT1-MMP, and TIMP-2 mRNAs.

## Results and Discussion

HSCs exhibited an in vivo morphology with long cellular processes when cultured on type I collagen gel. These results suggest that HSCs can recognize three-dimensional structure of extracellular type I collagen fibrils via cell surface integrin. Expression of MMP-1 protein and mRNA was detected by immunofluorescence staining and RT-PCR analysis, respectively, in HSCs cultured on type I collagen gel. The activity of MMP-1 was also detected by in situ zymography in cultured HSCs on type I collagen gel. These results indicate that the expression of MMP-1 was induced by interaction between extracellular type I collagen fibrils and cell surface integrin of cultured HSCs. Thus, the ECM components have regulatory effect on MMP-1 expression in cultured HSCs. Active form of MMP-2 was also detected by gelatin zymography in the conditioned medium of HSCs culture on type I collagen gel, but not on polystyrene surface, type I collagen-coated surface, or Matrigel.

**Figure 1:**
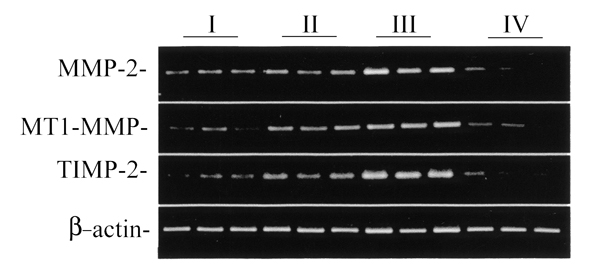
RT-PCR analysis of MMP-2, MT1-MMP, and TIMP-2 mRNAs in cultured HSCs. HSCs were cultured overnight on polystyrene (I), on type I collagen coated surface (II), on type I collagen gel (III), and on Matrigel (IV), respectively, and then the levels of MMP-2, MT1-MMP, and TIMP-2 mRNA were analysed by RT-PCR. The beta-actin mRNA level was used as internal standard.

**Figure 2:**
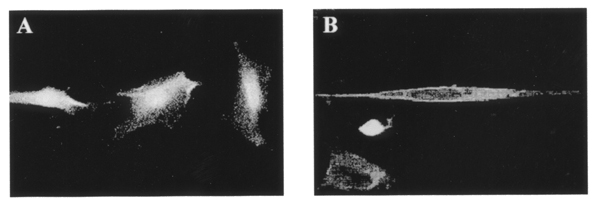
Immunofluorescence staining of MT1-MMP. HSCs were cultured overnight on polystyrene surface (A) or on type I collagen gel (B), and then stained for MT1-MMP proteins using monoclonal anti-MT1-MMP antibody.

Extracellular type I collagen fibrils were also found to elevate the expression of MMP-2 mRNA (Fig. 1). MMP-2 collagenase activity has been known to be tightly regulated by its relative stoichiometry with MT1-MMP and TIMP-2. Increased MT1-MMP protein was detected in cell membranes of HSCs cultured on type I collagen gel (Fig. 2).

MT1-MMP mRNA, and TIMP-2 mRNA expression (Fig. 1) were also increased in HSCs cultured on type I collagen gel, as indicated by RT-PCR analysis, suggesting the involvement of MT1-MMP and TIMP-2 in the processing of pro-MMP-2 to the active form. These results indicate that the expression of MMPs and TIMP-2 in cultured HSCs is regulated by ECM components, suggesting an important role of HSCs in the ECM remodeling of the liver tissue.
